# A low-cost and eco-friendly recombinant protein expression system using copper-containing industrial wastewater

**DOI:** 10.3389/fmicb.2024.1367583

**Published:** 2024-03-21

**Authors:** Xiaofeng Zhou, Qiyu Xiang, Yubei Wu, Yongjuan Li, Tiantian Peng, Xianxian Xu, Yongguang Zhou, Lihe Zhang, Jianghui Li, Linyong Du, Guoqiang Tan, Wu Wang

**Affiliations:** ^1^Key Laboratory of Laboratory Medicine, Ministry of Education of China, School of Laboratory Medicine and Life Science, Wenzhou Medical University, Wenzhou, Zhejiang, China; ^2^College of Life Science, Nanjing Agricultural University, Nanjing, Jiangsu, China; ^3^Department of Rheumatology, The Second Affiliated Hospital of Wenzhou Medical University, Wenzhou, China

**Keywords:** copper-induced, protein expression, industrial wastewater, low-cost, eco-friendly

## Abstract

The development of innovative methods for highly efficient production of recombinant proteins remains a prominent focus of research in the biotechnology field, primarily due to the fact that current commercial protein expression systems rely on expensive chemical inducers, such as isopropyl β-D-thiogalactoside (IPTG). In our study, we designed a novel approach for protein expression by creating a plasmid that responds to copper. This specialized plasmid was engineered through the fusion of a copper-sensing element with an optimized multiple cloning site (MCS) sequence. This MCS sequence can be easily customized by inserting the coding sequences of target recombinant proteins. Once the plasmid was generated, it was introduced into an engineered *Escherichia coli* strain lacking *copA* and *cueO*. With this modified *E. coli* strain, we demonstrated that the presence of copper ions can efficiently trigger the induction of recombinant protein expression, resulting in the production of active proteins. Most importantly, this expression system can directly utilize copper-containing industrial wastewater as an inducer for protein expression while simultaneously removing copper from the wastewater. Thus, this study provides a low-cost and eco-friendly strategy for the large-scale recombinant protein production. To the best of our knowledge, this is the first report on the induction of recombinant proteins using industrial wastewater.

## 1 Introduction

The expression of heterologous proteins in host organisms using genetic and protein engineering techniques are widely employed in various applications. Commercial recombinant protein expression plasmids currently require the addition of chemical inducers for efficient protein production, such as isopropyl β-D-thiogalactoside (IPTG) (Wang et al., 2018), arabinose (Guzman et al., 1995), lactose (Lutz and Bujard, 1997), methanol (Yang and Zhang, 2018). However, these reagents can be costly, especially in industrial-scale production of recombinant proteins. Although some auto-induction methods have been reported to eliminate the need for external inducers (Studier, 2005, 2014), they often necessitate specific self-inducing culture media, which can be cumbersome to prepare. Furthermore, the induction of recombinant protein bound with specific metals as cofactor also necessitates the addition of the corresponding metal solution to the culture medium.

Microorganisms have developed self-defense mechanisms to counteract the toxicity of heavy metals in the environment. For example, *E. coli* has evolved several copper efflux systems to survive when exposed to toxic levels of copper (Rensing and Grass, 2003). CueR is the transcriptional regulator of the *cue* system and can significantly promote the induction of *copA* and *cueO* upon copper exposure (Outten et al., 2000). CopA functions as a copper efflux pump that exports Cu^+^ from the cytoplasm to the periplasm (Rensing et al., 2000), and CueO is a copper oxidase that converts periplasmic Cu^+^ to the less toxic form Cu^2+^ (Grass and Rensing, 2001b). The *cue* system has recently been employed for the development of whole-cell biosensors and biosorbents to detect and remediate copper ions in water. *E. coli* cells harboring a vector with a simple fusion of the *copA* promoter (P*copA*) and a fluorescent protein-encoding gene were constructed as whole-cell biosensors to detect bioavailable copper ions (Kang et al., 2018). Additionally, *E. coli* was engineered to remove copper from actual environmental water samples through the adsorption facilitated by surface display of the CueR protein (Wang et al., 2019).

While numerous whole-cell biosensors have been developed for detecting heavy metal contaminants, limited attention has been given to the production of recombinant proteins based on heavy metal resistance/homeostasis mechanisms. In this study, we developed a method for efficient overexpression of recombinant proteins using copper ions as the inducer. Due to the sensitivity and robustness of this induction system, it is also applicable for inducing recombinant proteins using industrial wastewater containing copper ions. This method facilitates the efficient production of recombinant proteins by simply and directly adding copper-containing industrial wastewater to the cell culture, concomitantly removing copper from the wastewater. Notably, this approach stands out for its low cost and environmental friendliness. We applied this method to overexpress the copper-independent DNA polymerase Pfu and the copper-dependent laccase CueO protein, which are widely used in biological research and the food industry, respectively, thereby validating the advantages and applicability of this approach.

## 2 Materials and methods

### 2.1 Construction of expression vector and engineered *Escherichia coli* strain for protein induction by copper ions

The upstream promoter region of *E. coli copA* (−227 to −1 bp, P*copA*) (EcoGene accession number: EG13246) was fused to an optimized multiple cloning site (MCS) sequence, creating the P*copA*-MCS fragment synthesized by Genescript (Nanjing, China). For the initial construction of the copper-induced vector, P*copA*-MCS was amplified by PCR using primers P*copA*-MCS-1/P*copA*-MCS-2, and the linearized plasmid pUC57K (derived from pUC57, kanamycin resistant) was amplified by PCR using primers pUC57K-1/pUC57K-2. The P*copA*-MCS fragment was then ligated to the linearized pUC57K using the ClonFast kit based on seamless cloning technology (Messerschmidt et al., 2016), resulting in the copper-induced plasmid P*copA*-MCS-pUC57K. To induce *Pyrococcus furiosus* DNA polymerase Pfu (Enzyme Commission number:2.7.7.7) and the cytoplasmic part of *E. coli* multicopper oxidase CueO (Enzyme Commission number:1.16.3.4). DNA fragments encoding Pfu, and residues 29–516 of CueO, were codon-optimized, synthesized, and then amplified by PCR using primers Pfu-1/Pfu-2 and CueO-1/CueO-2, respectively. The obtained PCR products were subsequently cloned into the pre-linearized P*copA*-MCS-pUC57K, which had been digested by the restriction enzymes *Sal*I and *Hin*dIII. This cloning was also achieved using seamless cloning kit to generate two expression vectors: P*copA*-Pfu-pUC57K and P*copA*-CueO-pUC57K.

*Escherichia coli* cell lacking both *copA* and *cueO* (Δ*copA*/*cueO* double mutant) was genetically engineered from a wild-type *E. coli* strain (MC4100) as previously reported (Wang et al., 2019). The two obtained plasmids were then transformed into *E. coli* MC4100 and Δ*copA*/*cueO* strain, respectively, for subsequent protein expression.

All primers used are listed in Supplementary Table S1, the codon-optimized sequences of Pfu and CueO are present in Seq. S2 and Seq. S3, respectively, in the Supplementary material.

### 2.2 Protein expression of DNA polymerase Pfu and laccase CueO induced by copper solution

The strains P*copA*-Pfu-pUC57K/MC4100 and P*copA*-Pfu-pUC57K/Δ*copA*/*cueO* were cultured in LB medium at 37°C until the OD_600_ (optical density at 600 nm) reached 0.6. The culture was then divided into eight tubes and treated with CuCl_2_ solution at final concentrations of 0, 2, 5, 25, 100, 500, 1,000, 2000 μM at 16°C for 24 h. Afterward, cells were harvested, resuspended in Tris buffer (20 mM Tris–HCl, 500 mM NaCl, pH 8.0), and protein induction for each sample was detected by SDS-PAGE analysis. A similar treatment was conducted for the analysis of CueO expression using the cell strains P*copA*-CueO-pUC57K/MC4100 and P*copA*-CueO-pUC57K/Δ*copA*/*cueO*.

To test possible interference of other metal ions on the copper-induced expression of Pfu, Cu^2+^ (25 μM, final concentration) with each of the other nine metals (Mg^2+^, Ca^2+^, Fe^3+^, Zn^2+^, Co^2+^, Ni^2+^, Pb^2+^, As^5+^, Cr^6+^, at final concentration of 50 μM) were simultaneously added to the bacterial solutions before induction. The protein expression of each sample was analyzed by SDS-PAGE.

### 2.3 Protein expression of Pfu using copper-containing industrial wastewater

Water samples from electroplating rinsing effluents and the river were collected throughout Longwan industrial district in Wenzhou, Southeast China. The samples were centrifuged (12,000 rpm, 10 min) to remove any sediment. The strains P*copA*-Pfu-pUC57K/MC4100 and P*copA*-Pfu-pUC57K/Δ*copA*/*cueO* were cultivated in freshly prepared 2 × LB medium at 37°C until the OD_600_ reached 0.6. The bacterial culture was divided into several tubes. Each water sample containing excess copper ions was directly added to the same volume of the aforementioned two cell cultures, respectively. After 24 h of incubation at 16 C, the cells were harvested by centrifugation and the protein expression of each mixture was analyzed by SDS-PAGE. The produced protein was purified from the cells for further study.

### 2.4 Protein purification

To purify the protein, the culture was pelleted, and bacterial cells were resuspended in Tris buffer (20 mM Tris–HCl, 500 mM NaCl, pH 8.0) before being disrupted by a high-pressure homogenizer (JNBIO). The lysate was then centrifuged at 15,000 × *g* for 45 min, and the His_6_-tagged (6 × histidine tag) soluble proteins in the supernatant were purified using a Ni–agarose column (Qiagen co.), followed by a gel filtration column (SuperdexTM 75 10/300GL, GE), as described in Wang et al. (2014). The purity of the purified protein was analyzed using SDS/PAGE gel stained by Coomassie brilliant blue. The concentration of purified protein was determined using the Bradford Protein Assay Kit (Beyotime).

### 2.5 Activity assay of purified Pfu and CueO

The P*copA*-CueO-pUC57K plasmid was utilized as a template to assess the DNA polymerase activity of the purified Pfu protein. The forward and reverse primers used, P1 and P2, are listed in Supplementary Table S1. The PCR reaction mixture (50 μL) included 20 ng of the template plasmid, 5 μL of dNTPs (2.5 mM each), 0.5 μL of purified Pfu (at final concentrations of 3 μM, 1.5 μM, and 0.75 μM, respectively), 1 μL of each primer (20 μM), and the reaction buffer. Additionally, commercial Pfu enzyme (5 U/μL, Beyotime) was used as a positive control, and a reaction with no enzyme served as the negative control. Amplification was achieved with 35 cycles of denaturation for 40 s at 95°C, annealing for 40 s at 50°C, and extension for 4 min at 72°C. The reaction product was loaded onto a 1% agarose gel with staining dye and detected with visible light. The intensity of the DNA bands on the agarose gel was compared using the ImageJ software.

The analysis of CueO laccase activity was performed using ABTS (2,2′-azino-bis (3-ethylbenzthiazoline-6-sulfonic acid)) as the substrate (Zeng et al., 2011). The assay mixture (150 μL) consisted of 15 μL of CueO protein, 50 μL of ABTS (final concentration of 1 mM), and 85 μL of citrate buffer (pH 3.0). The mixture was incubated at 50°C for 20 min. For the negative control, an equivalent volume of buffer was used instead of protein. The absorbance at 420 nm (OD_420_) was measured, and the amount of product generated (μM) was calculated as (OD_420_/36) × 1,000 (ε_420_ = 36,000 M^−1^ cm^−1^). One unit (U) of enzyme activity was defined as the amount of the laccase required to oxidize 1 μmol of ABTS substrate per minute.

### 2.6 Determination of copper content in protein and wastewater sample

For the purified protein sample, 1 mL was mixed with 4 mL of HNO_3_ (35%) in a digestion vessel before undergoing microwave digestion. Following complete digestion, the sample was diluted by deionized water and the copper content of the digested sample was determined using inductively coupled plasma mass spectrometry (ICP-MS).

In the case of wastewater samples, whether before or after treatment by engineered *E. coli* cells, they were centrifuged (12,000 rpm, 10 min) to remove any insoluble particles. The supernatants were then mixed with a 4-fold volume of HNO_3_ (15%) before undergoing ICP-MS measurement.

## 3 Results

### 3.1 Construction of a protein expression system induced by copper ions

Typically, the fusion of the metal-sensing promoter region with a reporter element (encoding reporters like fluorescent proteins, β-galactosidase and luciferase) is employed to create bacterial cell-based biosensors for detecting specific metal ions (Hynninen and Virta, 2010). It can be hypothesized that by replacing the open reading frame (ORF) of the reporter gene with the coding sequence of the recombinant protein of interest (POI), while retaining the upstream promoter sequence, it becomes possible to induce the expression of recombinant protein in the presence of metal ions. Therefore, our goal was to develop an engineered bacterial system capable of inducing proteins using metal ions.

**Figure 1:**
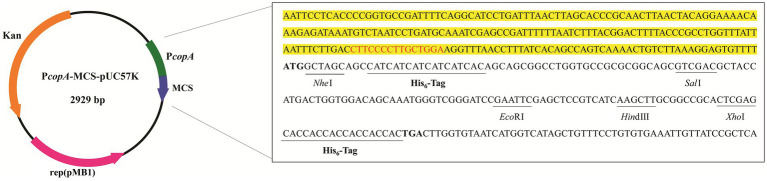
Schematic illustration of the vectors constructed for the copper-induced protein expression. The DNA sequence of the *E. coli copA* promoter region is highlighted in yellow, with the CueR binding site indicated by red letters. The optimized multiple cloning site (MCS) sequence commences with a bolded start codon (ATG) and ends with a bolded stop codon (TGA), incorporating two His_6_ coding sequences and several generally used restriction sites (underlined).

To test this concept, a plasmid capable of sensing copper ions was constructed by fusing the *E. coli copA* promoter to an optimized MCS sequence that contained several commonly used restriction sites. To facilitate subsequent protein purification by nickel affinity chromatography, two His_6_-tag coding sequences were included in the MCS sequence (Figure 1). The complete sequence of this generated copper-induced vector, P*copA*-MCS-pUC57K, is provided in Seq. S1 in the Supplementary material.

In general, to produce a POI with an N-terminal His_6_-tag, the gene coding sequence of the POI (*GOI*) with a stop codon can be inserted at *Sal*I or other downstream enzyme sites. To obtain a C-terminal His_6_-tagged POI, the *GOI* without a stop codon should be cloned into the plasmid double-digested by *Nhe*I and another enzyme, where the restriction site is located between the two His_6_-tag sequences (Figure 1).

### 3.2 Characterization of DNA polymerase Pfu protein expression induced by copper ions

**Figure 2:**
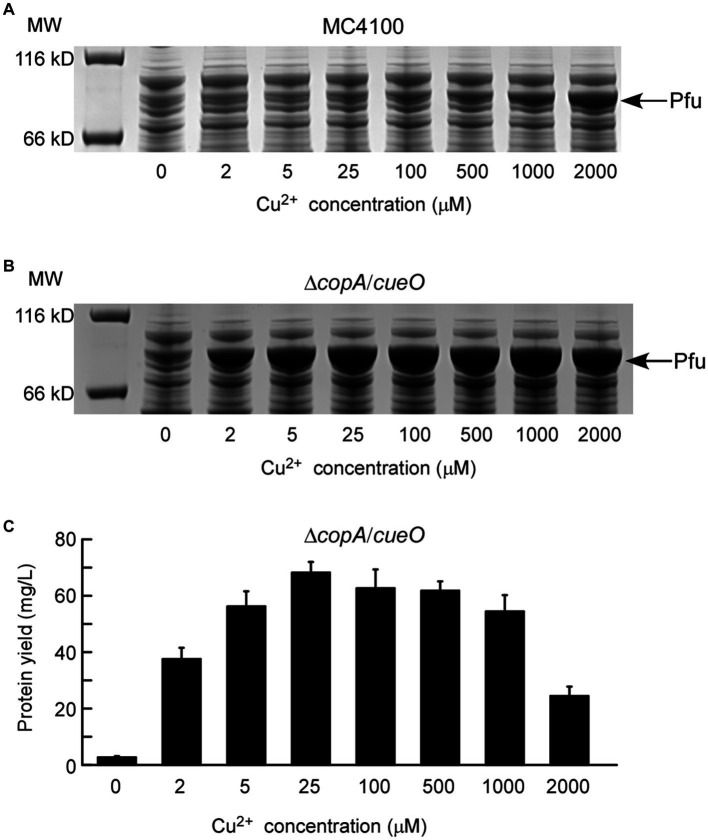
Characterization of protein expression of DNA polymerase Pfu induced by copper ions. The expressions of Pfu induced by increasing concentrations of copper ions in *E. coli* wild-type strain MC4100 **(A)** and Δ*copA*/*cueO* **(B)** were analyzed by whole-cell SDS-PAGE. The results are representative of three independent experiments. Panel **(C)** shows the determination of protein yield for the Pfu protein samples from **(B)**. Data are the means of three independent experiments, and error bars represent the standard deviation.

Initially, the applicability of the copper-induced protein expression system was assessed using Pfu DNA polymerase. Pfu is a widely used molecular enzyme derived from the hyperthermophilic *Pyrococcus furiosus*, employed for high-fidelity DNA synthesis in the polymerase chain reaction (Cline et al., 1996). An efficient and cost-effective Pfu expression method is essential for industrial-scale production. The copper-induced Pfu expression plasmid, P*copA*-Pfu-pUC57K, was introduced into *E. coli* MC4100, and cells were treated with varying concentrations of copper solutions. As depicted in Figure 2A, P*copA*-Pfu-pUC57K/MC4100 did not produce detectable Pfu protein until the Cu^2+^ concentration reached 1 mM, with only a slight protein band visible even at 2 mM Cu^2+^. Higher copper concentrations (> 2 mM) proved toxic to *E. coli* cells, impacting cell viability and protein synthesis (Results not shown). Recognizing *E. coli*’s intrinsic copper homeostatic systems, which maintain low intracellular copper content to mitigate copper toxicity (Rensing and Grass, 2003), we addressed this by employing the Δ*copA*/*cueO* double mutant strain. This strain, hypersensitive to copper, showed significant Pfu protein yield with increasing Cu^2+^ concentrations (2–2,000 μM) (Figure 2B). Remarkably, the addition of 2 μM copper ions sufficed for substantial protein production. The maximum protein yield of Pfu was approximately 68.23 mg/L (68.23 mg purified protein obtained from 1 L of bacterial culture) after incubation with 25 μM Cu^2+^ at 16°C for 24 h (Figure 2C), surpassing yields achieved with previous methods (Dabrowski and Kur, 1998; Zheng et al., 2016).

**Figure 3:**
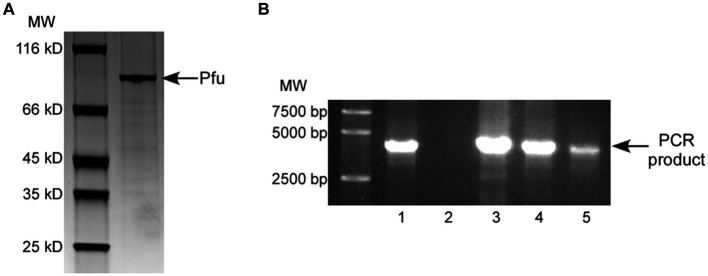
**(A)** SDS-PAGE of the purified Pfu polymerase prepared from the *E. coli* Δ*copA*/*cueO* cells induced by 25 μM Cu^2+^ solution. **(B)** Estimation of enzymatic activity for the purified Pfu polymerase from **(A)** by PCR. The purified Pfu from the stock was diluted in PCR buffer to final concentrations of 3 (Lane 3), 1.5 (Lane 4), and 0.75 μM (Lane 5) respectively. The PCR amplification was run in parallel with the similar amount of Pfu DNA polymerase from Beyotime (Lane 1, 5 U/μL). A sample contained no enzyme was used as a negative control (Lane 2). The results are representative of three independent experiments.

To assess the DNA polymerase activity of Pfu, the N-terminal His_6_-tagged protein was purified from Δ*copA*/*cueO* cells treated with 25 μM Cu^2+^ (purity >95%, as shown in Figure 3). Enzymatic activity of Pfu DNA polymerase induced by copper ions was estimated based on band intensity matching of the PCR product to that of commercial Pfu DNA polymerase. Based on the relative intensity of the bands in the agarose gel, 0.5 μL of diluted purified recombinant Pfu (Figure 3B, Lane 4, protein concentration was adjusted to about 1.5 μM, similar to that of commercial Pfu) showed an activity of equivalent to 5 U/μL of commercial Pfu (Figure 3B, Lane 1). The specific activity of Pfu was calculated to be 36,122 U/mg, which was comparable to previous reports (Zheng et al., 2016; Chen et al., 2023).

In addition, the Pfu protein samples purified from the Δ*copA*/*cueO* cells treated with copper contained negligible amounts of copper, even when exposed to 2,000 μM copper ions (about 0.028 molecules of copper per Pfu monomer). Therefore, the copper ions in the culture medium would not affect the activity of Pfu DNA polymerase.

**Figure 4:**
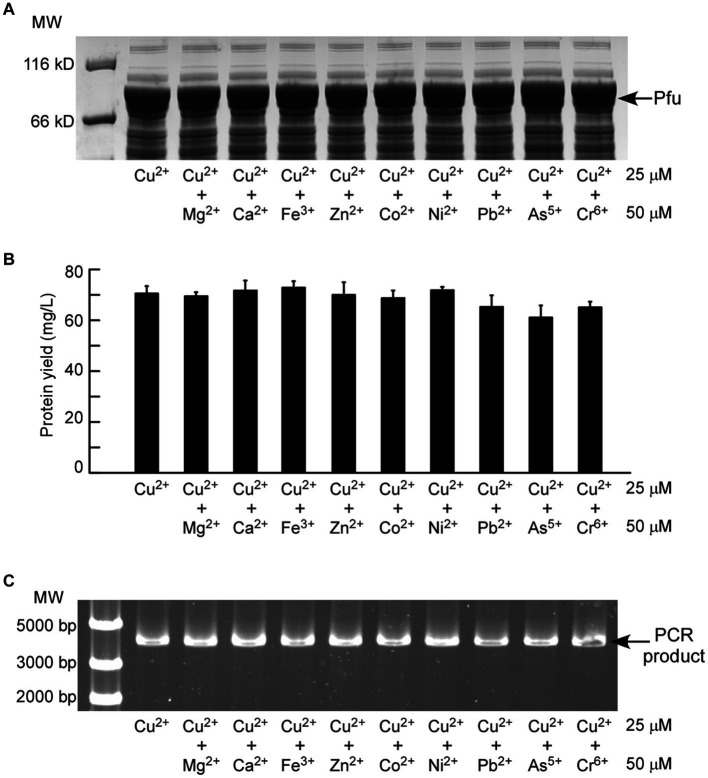
**(A)** SDS-PAGE analysis showing the effect of other heavy metals on the copper-induced expression of Pfu in Δ*copA*/*cueO*. The protein yield **(B)** and enzymatic activity **(C)** of the Pfu protein samples purified from **(A)** were compared. The results presented are representative of three independent experiments.

Due to the high cost of laboratory-grade copper reagent, we explored the feasibility of using copper-containing mixtures (e.g., industrial wastewater) as the inducer for protein expression. Because of the complexity of wastewater samples, which may contain various compounds, we tested whether the copper-induced protein expression was susceptible to other metals. The results demonstrated that copper-induced protein expression was not significantly affected even when a 2-fold excess of each metal was added along with 25 μM Cu^2+^ (Figures 4A,B). Furthermore, the DNA polymerase activity of Pfu purified from these samples showed no notable alteration (Figure 4C). These results collectively illustrate that the protein induction efficiency of this expression system and the activity of the induced Pfu protein remain unaffected by other metals.

### 3.3 Characterization of copper-dependent laccase CueO protein expression induced by copper ions

Laccases, enzymes containing multiple copper atoms, exhibit the ability to oxidize a broad range of substrates. Laccases can be utilized as versatile biocatalysts that have been used in different industrial fields, including paper, textile, food processing and environmental protection (Upadhyay et al., 2016). The *E. coli* laccase CueO, part of the copper regulatory *cue* operon, is responsible for oxidizing Cu^+^ to the less harmful Cu^2+^ in the periplasm. The analyses of the crystal structures of CueO bound to Cu^+^ provided evidence that the methionine-rich region binds Cu^+^ and oxidizes it to Cu^2+^ (Singh et al., 2011). Apart from alleviation of copper cytotoxicity, CueO also demonstrates a relatively broad substrate spectrum, similar to laccases from other species. As a typical multicopper oxidase, CueO possesses four copper atoms per monomer (Grass and Rensing, 2001a) that together are essential for its oxidase activity (Ueki et al., 2006). Hence, in addition to conventional inducer, the addition of excess copper ions is required for the preparation of active CueO (Li et al., 2007; Decembrino et al., 2021).

**Figure 5:**
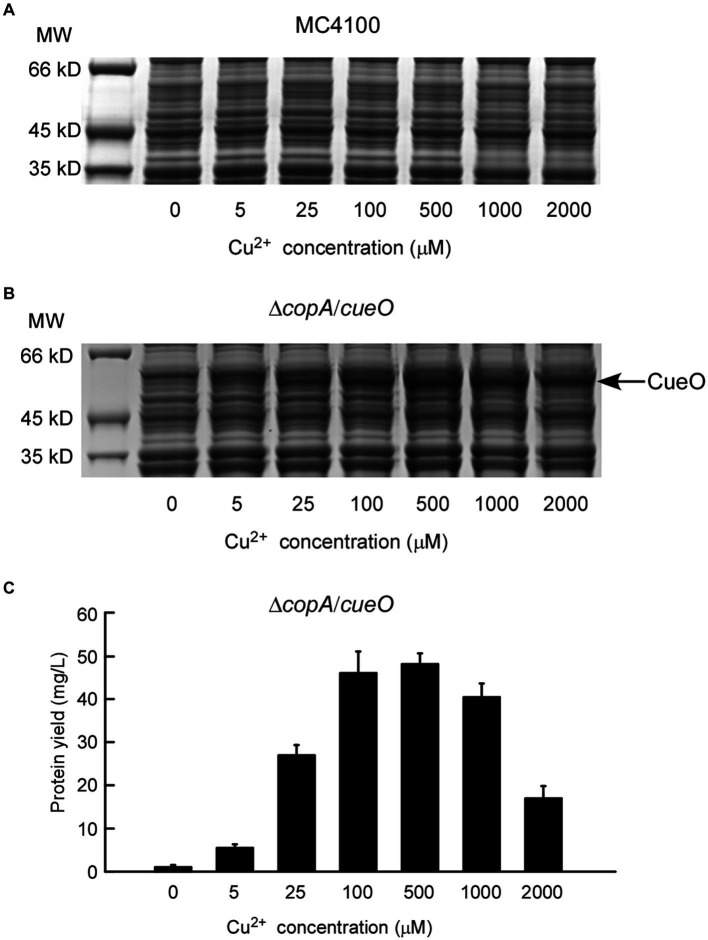
Characterization of protein expression of *E. coli* laccase CueO induced by copper ions. The expressions of CueO induced by increasing concentrations of copper ions in *E. coli* wild strain MC4100 **(A)** and Δ*copA*/*cueO* **(B)** were analyzed by whole-cell SDS-PAGE. The results are representative of three independent experiments. Panel **(C)** shows the determination of protein yield for the CueO protein samples from **(B)**. Data are the means of three independent experiments, and error bars represent the standard deviation.

To explore the production of the active form of the copper-binding protein using this expression system, the constructed p*copA*-CueO-pUC57K was transformed into MC4100 and Δ*copA*/*cueO* strains, respectively. The cells were incubated with increasing amounts of Cu^2+^ before harvest for protein induction analysis. As shown on the whole-cell SDS-PAGE gel, CueO in Δ*copA*/*cueO* was gradually induced as the exogenous Cu^2+^ concentration in the medium increased from 0 to 500 μM (Figure 5B). In line with the Pfu induction result (Figure 2A), CueO expression in MC4100 under the same experimental conditions was minimal (Figure 5A). The maximum protein yield of CueO was about 47.94 mg/L when treated with 500 μM Cu^2+^. However, higher copper concentrations (1 mM and 2 mM) decreased CueO expression, likely due to the hypersensitivity of the Δ*copA*/*cueO* strain to copper ions (Figure 5C).

**Figure 6:**
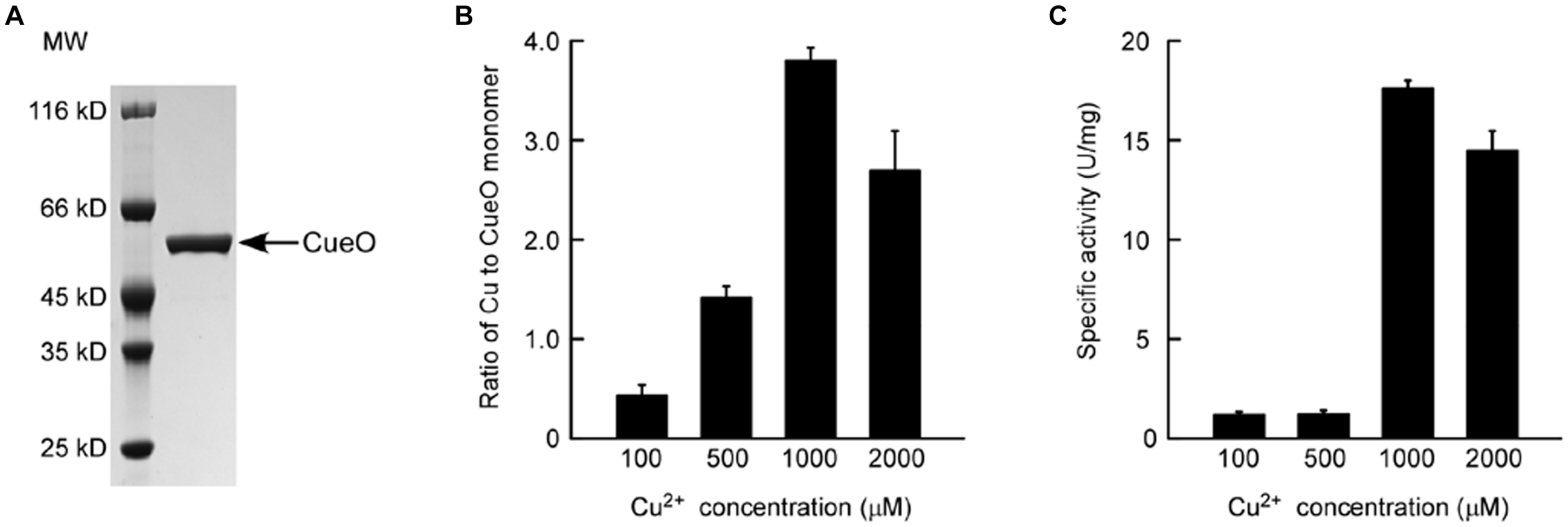
SDS-PAGE analysis of the purified CueO prepared from *E. coli* Δ*copA*/*cueO* cells induced by **(A)** 500 μM Cu^2+^ solution. **(B)** Determination of copper content in the CueO protein samples induced by increasing concentrations of Cu^2+^ solutions using ICP-MS. **(C)** Oxidase activity analysis of the copper-induced CueO protein samples. Data are the means of three independent experiments, and error bars represent the standard deviation.

Subsequently, the N-terminal His_6_-tagged CueO protein (signal peptide of residues 1–28 was excluded to prevent export to the periplasm) induced by different concentrations of copper ions was purified from Δ*copA*/*cueO* for activity assay and copper content measurement (Figure 6A). The results in Figure 6B revealed that the CueO protein sample induced by low concentration (5 or 25 μM) of Cu^2+^ did not contain any detectable copper atoms, while the copper content in CueO dramatically increased with Cu^2+^ concentration ranging from 100 to 1,000 μM. The copper content appeared to be nearly saturated (approximately 3.8 molecules of copper per CueO monomer) when the concentration reached 1,000 μM. This result indicates that a modest amount of copper is sufficient for efficient protein expression, while excess copper is necessary for the binding of copper to the protein. Unexpectedly, with the Cu^2+^ concentration up to 2,000 μM, the copper content in CueO decreased, possibly due to protein misfolding caused by an overload of intracellular copper ions. The oxidase activity of CueO was substantially correlated with the amount of copper bound in the protein (Figure 6C), consistent with previous reports indicating that the activity is greatly enhanced in the presence of excess copper ions (Kim et al., 2001; Roberts et al., 2002). Previous studies have suggested that CueO contains four Cu atoms: a type I copper (T1 Cu) and a trinuclear copper center (TNC) consisting of a type II copper (T2 Cu) and a pair of type III coppers (T3 Cu) (Grass and Rensing, 2001a; Komori et al., 2012). Based on the analysis of crystal structure, the regulatory T1 copper, has a catalytic role in the protein, but is buried under a methionine-rich helix that limits access to substrate. Thus, the coordination of copper ions in TNC would significantly enchance the oxidase activity (Roberts et al., 2002). Figure 6 shows that CueO purified from cells treated with 500 μM Cu^2+^ contained about 1.4 molecules of copper per monomer (Figure 6B) but exhibited very low activity (Figure 6C), further demonstrating that a sufficient amount of copper is necessary for the oxidase activity of CueO.

### 3.4 Application of protein expression induced by copper-containing industrial wastewater

Copper, a toxic heavy metal present in industrial wastewaters, poses a severe threat to human health and ecosystems when continuously discharged into the environment. Because physicochemical methods are energy- and cost-intensive and result in the generation of secondary pollution, bioremediation techniques have gained increasing attention. Given the potential for recombinant protein induction by copper ions, we aimed to investigate whether copper ions in industrial sewage could be utilized for protein induction while concurrently removed from wastewater.

**Figure 7:**
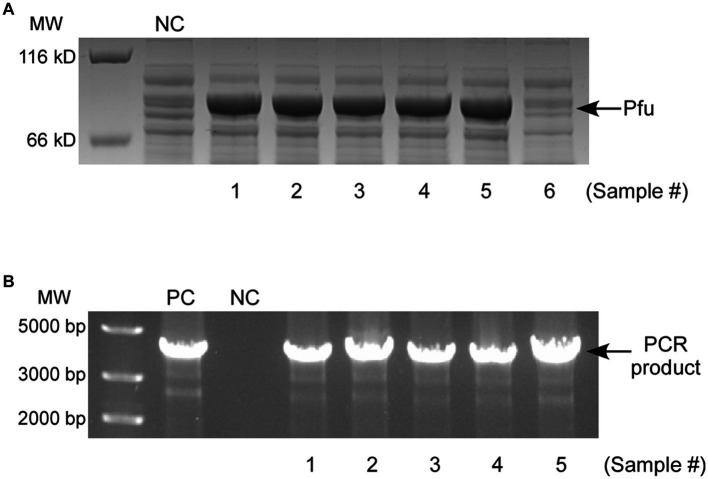
**(A)** SDS-PAGE analysis of Pfu expression in Δ*copA*/*cueO* induced by five copper-containing industrial wastewater samples (Lane 3–7) and a river water sample (Lane 8). The negative control (NC, Lane 2) is the sample induced by distilled water. **(B)** Enzymatic activity analysis of the purified Pfu proteins from **(A)** (Lane 4–8). The positive control (PC, Lane 2) and negative control (NC, Lane 3) represent the PCR samples using commercial polymerase and no enzyme, respectively. The results are representative of three independent experiments.

To evaluate the applicability of copper-induced protein expression system for wastewater samples, testing was conducted on five randomly selected samples collected from industrial sewage, each containing more than 1.5 mg/L of copper (Table 1), as determined by ICP-MS (Note: 1.5 mg/L is the allowable copper content in drinking water recommended by the World Health Organization). As the induction of active CueO required a relatively high level of Cu^2+^ (Figure 6C), DNA polymerase Pfu was chosen for verification. Similar to conventional inducer usage, the wastewater samples were directly mixed with bacterial growth medium, with an equal volume of wastewater to culture medium. All five samples significantly induced Δ*copA*/*cueO* cells to produce Pfu protein (Figure 7A, Lane 3–7) that proved effective in DNA amplification (Figure 7B, Lane 4–8). The river sample, containing negligible copper, failed to induce Pfu expression (Figure 7A, Lane 8). However, there was almost no protein induced in MC4100 treated with the same wastewater sample (results not shown), consistent with the results using laboratory-prepared copper solution (Figure 2). These findings demonstrate the effectiveness and robustness of our developed system in expressing recombinant protein using copper-containing industrial wastewater, even in the presence of moderate acidity (Note: The LB medium provided very strong buffering capability and could neutralize acid wastewater samples to pH values between 6 and 7) and high salinity, as indicated by pH and TDS (Table 1).

**Table 1:** Quantification of copper ions in wastewater samples before and after treatment with the copper-induced systems in *E. coli* MC4100 and Δ*copA*/*cueO*.

Samples	1	2	3	4	5	6
^b^pH	5.2	4.1	5.8	6.1	3.5	7.8
^a^pH	6.5	6.4	6.5	6.5	6.4	6.6
^*^TDS (g/L)	1.06	3.03	1.85	2.84	4.47	0.82
^b^Cu^2+^ (mg/L)	2.94	4.16	1.54	2.43	5.31	0.004
^a1^Cu^2+^ (mg/L)	1.24	3.27	0.77	1.09	3.36	0.001
^a2^Cu^2+^ (mg/L)	2.50	3.85	1.21	2.16	4.52	0.001

The samples 1–5 were industrial wastewater samples, and the sample 6 was a river water sample. The Cu^2+^ concentrations of all the samples were determined by ICP-MS.

^b^pH: the pH value of the originally collected sample.

^a^pH: the pH value of the cell culture mixed with collected sample.

^*^TDS: total dissolved solids.

^b^Cu^2+^: Cu^2+^ concentration of sample before treatment.

^a1^Cu^2+^: Cu^2+^ concentration of sample after treatment with P*copA*-Pfu-pUC57K/Δ*copA*/*cueO*.

^a2^Cu^2+^: Cu^2+^ concentration of sample after treatment with P*copA*-Pfu-pUC57K/MC4100.

Furthermore, after treatment with engineered cells, the copper content of wastewater samples was re-quantified by ICP-MS. Following incubation with cell culture of Δ*copA*/*cueO*, the average copper concentration of all wastewater samples decreased to about half (55.8 ± 15.1%) of the original content, while only 14.0 ± 5.2% of the total copper was removed by MC4100 (Table 1). This result can be attributed to the deletion of *copA* and *cueO*, which was previously proposed to enhance intracellular copper accumulation (Rensing et al., 2000). Among the five tested samples, three were detoxified to safe levels (< 1.5 mg/L) by Δ*copA*/*cueO*, as analyzed by ICP-MS. The other two samples still contained over-limit levels of copper (Table 1), likely due to the excessive copper content that could not be adequately removed by either MC4100 or Δ*copA*/*cueO*. Nevertheless, these results highlight the dual-functionality of our system: while inducing protein expression, it also has the potential to simultaneously remediate copper ions in the wastewater.

## 4 Discussion

Ensuring high-yield production of recombinant proteins is of utmost significance for both fundamental research and practical applications. Currently, numerous commercial expression systems are available. However, some of these systems are deemed impractical for large-scale production due to the high costs associated with prerequisite inducers. Continuous efforts have been dedicated to the development and optimization of protein expression approaches. Although various novel inducible expression systems have been reported using inducers such as uric acid (Liang et al., 2015) and iron-chelator (Lim et al., 2008), as well as physical methods like temperature (Qing et al., 2004) and light (Lalwani et al., 2021), these systems were just designed to address specific circumstances and may not be suitable for general applications. A recent study introduced a copper-induced expression system in *E. coli*, which was based on the combination of the two-component *cus* system and T7 RNA polymerase. This system successfully induced a substantial amount of protocatechuic acid using copper solution under optimal experimental conditions (Liu et al., 2023). However, the reagent-grade copper solution remains relatively expensive. Therefore, our goal was to explore the possibility of utilizing zero-cost copper-containing industrial sewage as an alternative to expensive pure copper solutions. Given that engineered bacterial cells can partially remove excess copper ions while inducing protein expression, our protein expression system offers both economic and ecological benefits.

The primary challenge in directly using wastewater is the presence of complex impurities in untreated industrial sewage. Fortunately, *E. coli* has evolved to acquire various resistance systems against external stresses, enabling cells to survive in extreme environments such as industrial wastewater. Additionally, the nutritional ingredients in the LB rich medium may bind to hazardous components, thereby attenuating the toxicity and acidity of wastewater to bacteria.

Secondly, while the copper-induced expression system is theoretically applicable for the production of any recombinant protein, additional optimization may be required for specific proteins. For instance, the low temperature (16°C) and sufficient induction time (24 h) used for protein production in this study likely contributed to the enhanced protein yield, as expression at a low temperature for a sufficient induction time is a well-known technique to avoid or limit aggregation of the recombinant protein and yield more soluble and well-folded protein (Kaur and Kumar, 2018).

Furthermore, it has been suggested that copper and other metals may have a similar ligand binding coordination in some metalloproteins. For example, excess copper could compete with iron for the metal binding site in IscA, thereby inhibiting iron–sulfur assembly in *E. coli* (Tan et al., 2014). The iron–sulfur clusters of dehydratases were found to be primary targets of copper ions, which could damage these proteins by liganding to the coordinating sulfur atoms (Macomber and Imlay, 2009). In such cases, while ensuring the protein yield, lowering the Cu^2+^ concentration would be helpful to avoid potential interference caused by excess copper. Another useful strategy is to develop additional protein expression systems induced by other metals using similar approaches, thanks to the well-demonstrated homeostasis and resistance mechanisms of various metals in bacteria. We have established a zinc-induced expression system for recombinant protein production based on the *znt* operon. It has proven effective when using zinc-containing wastewater, and particularly advantageous for the production of zinc-binding proteins (unpublished work). Although further studies are needed before this protein expression system can be widely applied on a large scale, our present work provides a promising approach for low-cost and eco-friendly protein production.

## Data availability statement

The original contributions presented in the study are included in the article/Supplementary material, further inquiries can be directed to the corresponding authors.

## Author contributions

XZ: Investigation, Methodology, Writing – original draft. QX: Investigation, Methodology, Writing – original draft. YW: Investigation, Methodology, Writing – original draft. YL: Investigation, Methodology, Writing – original draft. TP: Investigation, Methodology, Writing – original draft. XX: Investigation, Methodology, Writing – original draft. YZ: Investigation, Methodology, Writing – original draft. LZ: Investigation, Methodology, Writing – original draft. JL: Investigation, Methodology, Writing – original draft. LD: Investigation, Methodology, Writing – original draft, Writing – review & editing. GT: Conceptualization, Writing – original draft, Writing – review & editing. WW: Conceptualization, Funding acquisition, Investigation, Methodology, Supervision, Validation, Writing – original draft, Writing – review & editing.
