# Supplementary material for: A low-cost and eco-friendly recombinant protein expression system using copper-containing industrial wastewater
Source: Front Microbiol. 2024 Mar 21;15:1367583. doi: 10.3389/fmicb.2024.1367583 (PMC10995868; doi:10.3389/fmicb.2024.1367583)
Supplement: Supplementary file 1 [file Table_1.DOCX]

Supplementary Material

A low-cost and eco-friendly recombinant protein expression system using copper-containing industrial wastewater

**Xiaofeng Zhou^1^, Qiyu Xiang^2^, Yubei Wu^1^, Yongjuan Li^1^, Tiantian Peng^1^, Xianxian Xu^1^, Yongguang Zhou^1^, Yanwen Yu^1^, Lihe Zhang^3^, Jianghui Li^1^, Linyong Du^1*^, Guoqiang Tan^1*^, Wu Wang^1*^**

^1^Key Laboratory of Laboratory Medicine, Ministry of Education of China, School of Laboratory Medicine and Life Science, Wenzhou Medical University, Wenzhou, Zhejiang, China

^2^Country College of Life Science, Nanjing Agricultural University, Nanjing, Jiangsu, China

^3^Department of Rheumatology, The Second Affiliated Hospital of Wenzhou Medical University, Wenzhou, China

*** Correspondence:**Wu Wang

wangwu@wmu.edu.cn

Guoqiang Tan

tgq@wmu.edu.cn

Linyong Du

dulinyong@qq.com

# Supplementary Tables

**Table S1** Primers used for construction of P*copA*-MCS-pUC57K, P*copA*-Pfu-pUC57K, P*copA*-CueO-pUC57K and that used for DNA polymerase activity analysis of the purified Pfu protein

| Primer name | Primer Sequence（5′→3′） |
| --- | --- |
| P*copA*-MCS-1 | AATTCCTCACCCCGGTGCCG |
| P*copA*-MCS-2 | AAGTCAGTGGTGGTGGTGGT |
| pUC57K-1 | ACCACCACCACCACTGACTTGGTGTAATCATGGTCATAGCTG |
| pUC57K-2 | CGGCACCGGGGTGAGGAATTGATATCTAGATGTATTCGCGAGGTAC |
| Pfu-1 | GTGCCGCGCGGCAGCGTCGACATGATCCTGGACGTGGACTAC |
| Pfu-2 | CTCGAGTGCGGCCGCAAGCTTTTAGCTTTTCTTGATGTTCAGCCAGC |
| CueO-1 | GTGCCGCGCGGCAGCGTCGACATGGCTGAAAGGCCTACACTACCC |
| CueO-2 | CTCGAGTGCGGCCGCAAGCTTTCACACGGTAAAGCCCAGC |
| P1 | GGTGATGACGGTGAAAACCTCTGAC |
| P2 | CAATCTATCGCTTGTATGGGAAGCCCG |

# Supplementary DNA sequences

## Sequence S1 The complete DNA sequences of P*copA*-MCS-pUC57K

TCGCGCGTTTCGGTGATGACGGTGAAAACCTCTGACACATGCAGCTCCCGGAGACGGTCACAGCTTGTCTGTAAGCGGATGCCGGGAGCAGACAAGCCCGTCAGGGCGCGTCAGCGGGTGTTGGCGGGTGTCGGGGCTGGCTTAACTATGCGGCATCAGAGCAGATTGTACTGAGAGTGCACCATATGCGGTGTGAAATACCGCACAGATGCGTAAGGAGAAAATACCGCATCAGGCGCCATTCGCCATTCAGGCTGCGCAACTGTTGGGAAGGGCGATCGGTGCGGGCCTCTTCGCTATTACGCCAGCTGGCGAAAGGGGGATGTGCTGCAAGGCGATTAAGTTGGGTAACGCCAGGGTTTTCCCAGTCACGACGTTGTAAAACGACGGCCAGAGAATTCGAGCTCGGTACCTCGCGAATACATCTAGATATCAATTCCTCACCCCGGTGCCGATTTTCAGGCATCCTGATTTAACTTAGCACCCGCAACTTAACTACAGGAAAACAAAGAGATAAATGTCTAATCCTGATGCAAATCGAGCCGATTTTTTAATCTTTACGGACTTTTACCCGCCTGGTTTATTAATTTCTTGACCTTCCCCTTGCTGGAAGGTTTAACCTTTATCACAGCCAGTCAAAACTGTCTTAAAGGAGTGTTTTATGGCTAGCAGCCATCATCATCATCATCACAGCAGCGGCCTGGTGCCGCGCGGCAGCGTCGACGCTACCATGACTGGTGGACAGCAAATGGGTCGGGATCCGAATTCGAGCTCCGTCATCAAGCTTGCGGCCGCACTCGAGCACCACCACCACCACCACTGACTTGGTGTAATCATGGTCATAGCTGTTTCCTGTGTGAAATTGTTATCCGCTCACAATTCCACACAACATACGAGCCGGAAGCATAAAGTGTAAAGCCTGGGGTGCCTAATGAGTGAGCTAACTCACATTAATTGCGTTGCGCTCACTGCCCGCTTTCCAGTCGGGAAACCTGTCGTGCCAGCTGCATTAATGAATCGGCCAACGCGCGGGGAGAGGCGGTTTGCGTATTGGGCGCTCTTCCGCTTCCTCGCTCACTGACTCGCTGCGCTCGGTCGTTCGGCTGCGGCGAGCGGTATCAGCTCACTCAAAGGCGGTAATACGGTTATCCACAGAATCAGGGGATAACGCAGGAAAGAACATGTGAGCAAAAGGCCAGCAAAAGGCCAGGAACCGTAAAAAGGCCGCGTTGCTGGCGTTTTTCCATAGGCTCCGCCCCCCTGACGAGCATCACAAAAATCGACGCTCAAGTCAGAGGTGGCGAAACCCGACAGGACTATAAAGATACCAGGCGTTTCCCCCTGGAAGCTCCCTCGTGCGCTCTCCTGTTCCGACCCTGCCGCTTACCGGATACCTGTCCGCCTTTCTCCCTTCGGGAAGCGTGGCGCTTTCTCATAGCTCACGCTGTAGGTATCTCAGTTCGGTGTAGGTCGTTCGCTCCAAGCTGGGCTGTGTGCACGAACCCCCCGTTCAGCCCGACCGCTGCGCCTTATCCGGTAACTATCGTCTTGAGTCCAACCCGGTAAGACACGACTTATCGCCACTGGCAGCAGCCACTGGTAACAGGATTAGCAGAGCGAGGTATGTAGGCGGTGCTACAGAGTTCTTGAAGTGGTGGCCTAACTACGGCTACACTAGAAGAACAGTATTTGGTATCTGCGCTCTGCTGAAGCCAGTTACCTTCGGAAAAAGAGTTGGTAGCTCTTGATCCGGCAAACAAACCACCGCTGGTAGCGGTGGTTTTTTTGTTTGCAAGCAGCAGATTACGCGCAGAAAAAAAGGATCTCAAGAAGATCCTTTGATCTTTTCTACGGGGTCTGACGCTCAGTGGAACGAAAACTCACGTTAAGGGATTTTGGTCATGAGATTATCAAAAAGGATCTTCACCTAGATCCTTTTAAATTAAAAATGAAGTTTTAAATCAAGCCCAATCTGAATAATGTTACAACCAATTAACCAATTCTGATTAGAAAAACTCATCGAGCATCAAATGAAACTGCAATTTATTCATATCAGGATTATCAATACCATATTTTTGAAAAAGCCGTTTCTGTAATGAAGGAGAAAACTCACCGAGGCAGTTCCATAGGATGGCAAGATCCTGGTATCGGTCTGCGATTCCGACTCGTCCAACATCAATACAACCTATTAATTTCCCCTCGTCAAAAATAAGGTTATCAAGTGAGAAATCACCATGAGTGACGACTGAATCCGGTGAGAATGGCAAAAGTTTATGCATTTCTTTCCAGACTTGTTCAACAGGCCAGCCATTACGCTCGTCATCAAAATCACTCGCATCAACCAAACCGTTATTCATTCGTGATTGCGCCTGAGCGAGACGAAATACGCGATCGCTGTTAAAAGGACAATTACAAACAGGAATCGAATGCAACCGGCGCAGGAACACTGCCAGCGCATCAACAATATTTTCACCTGAATCAGGATATTCTTCTAATACCTGGAATGCTGTTTTTCCGGGGATCGCAGTGGTGAGTAACCATGCATCATCAGGAGTACGGATAAAATGCTTGATGGTCGGAAGAGGCATAAATTCCGTCAGCCAGTTTAGTCTGACCATCTCATCTGTAACATCATTGGCAACGCTACCTTTGCCATGTTTCAGAAACAACTCTGGCGCATCGGGCTTCCCATACAAGCGATAGATTGTCGCACCTGATTGCCCGACATTATCGCGAGCCCATTTATACCCATATAAATCAGCATCCATGTTGGAATTTAATCGCGGCCTCGACGTTTCCCGTTGAATATGGCTCATAACACCCCTTGTATTACTGTTTATGTAAGCAGACAGTTTTATTGTTCATGATGATATATTTTTATCTTGTGCAATGTAACATCAGAGATTTTGAGACACGGGCCAGAGCTGCA

## Sequence S2 The codon-optimized DNA sequence of Pfu

ATGATCCTGGACGTGGACTACATTACCGAAGAGGGCAAGCCGGTTATCCGCCTGTTCAAGAAAGAGAATGGCAAGTTCAAAATCGAGCACGACCGTACCTTCCGTCCGTACATCTATGCGCTGCTGCGTGACGATAGCAAAATTGAGGAAGTGAAGAAAATCACCGGCGAGCGTCACGGCAAGATTGTGCGTATCGTGGACGTTGAAAAAGTTGAGAAGAAATTTCTGGGTAAACCGATTACCGTTTGGAAGCTGTACCTGGAACACCCGCAGGATGTTCCGACCATCCGTGAGAAAGTGCGTGAACACCCGGCGGTGGTTGACATTTTCGAGTACGATATCCCGTTTGCGAAACGTTATCTGATTGACAAGGGTCTGATCCCGATGGAAGGCGAGGAAGAGCTGAAAATTCTGGCGTTCGATATCGAAACCCTGTATCACGAAGGCGAAGAGTTTGGCAAGGGCCCGATCATTATGATTAGCTACGCGGACGAGAACGAAGCGAAAGTGATTACCTGGAAGAACATCGATCTGCCGTACGTTGAGGTGGTTAGCAGCGAGCGTGAAATGATCAAGCGTTTCCTGCGTATCATTCGTGAAAAAGACCCGGATATCATTGTGACCTACAACGGTGACAGCTTCGATTTTCCGTATCTGGCGAAGCGTGCGGAGAAACTGGGCATTAAGCTGACCATCGGTCGTGACGGCAGCGAGCCGAAGATGCAGCGTATTGGTGATATGACCGCGGTGGAAGTTAAAGGCCGTATCCACTTCGACCTGTATCACGTGATTACCCGTACCATCAACCTGCCGACCTACACCCTGGAGGCGGTGTATGAAGCGATTTTTGGTAAACCGAAGGAGAAAGTTTACGCGGACGAAATCGCGAAAGCGTGGGAAAGCGGCGAGAACCTGGAACGTGTTGCGAAATACAGCATGGAGGATGCGAAGGCGACCTATGAGCTGGGTAAAGAATTCCTGCCGATGGAAATCCAGCTGAGCCGTCTGGTTGGTCAACCGCTGTGGGATGTGAGCCGTAGCAGCACCGGCAACCTGGTGGAGTGGTTTCTGCTGCGTAAGGCGTACGAGCGTAACGAAGTTGCGCCGAACAAACCGAGCGAAGAGGAATACCAACGTCGTCTGCGTGAGAGCTATACCGGTGGCTTCGTGAAAGAGCCGGAAAAGGGTCTGTGGGAAAACATCGTTTACCTGGACTTTCGTGCGCTGTATCCGAGCATCATTATCACCCACAACGTGAGCCCGGACACCCTGAACCTGGAAGGTTGCAAAAACTATGATATCGCGCCGCAGGTTGGCCACAAGTTCTGCAAAGATATTCCGGGTTTTATTCCGAGCCTGCTGGGTCACCTGCTGGAGGAACGTCAGAAGATTAAAACCAAGATGAAAGAAACCCAAGACCCGATTGAAAAGATCCTGCTGGATTACCGTCAAAAGGCGATCAAACTGCTGGCGAACAGCTTCTACGGTTACTATGGCTATGCGAAAGCGCGTTGGTATTGCAAAGAATGCGCGGAAAGCGTGACCGCGTGGGGTCGTAAGTACATTGAGCTGGTTTGGAAAGAACTGGAGGAAAAATTCGGTTTTAAGGTGCTGTACATCGACACCGATGGCCTGTATGCGACCATTCCGGGTGGCGAGAGCGAGGAAATCAAGAAAAAGGCGCTGGAATTCGTTAAATATATTAACAGCAAGCTGCCGGGCCTGCTGGAGCTGGAATACGAGGGTTTTTATAAACGTGGCTTCTTTGTTACCAAAAAGCGTTACGCGGTGATCGACGAGGAAGGTAAAGTGATTACCCGTGGCCTGGAGATCGTGCGTCGTGATTGGAGCGAGATTGCGAAGGAAACCCAGGCGCGTGTGCTGGAAACCATCCTGAAACACGGTGACGTTGAGGAAGCGGTGCGTATTGTTAAAGAAGTGATCCAGAAGCTGGCGAACTACGAGATCCCGCCGGAAAAGCTGGCGATTTATGAGCAAATCACCCGTCCGCTGCACGAATACAAAGCGATTGGTCCGCACGTGGCGGTTGCGAAAAAGCTGGCGGCGAAGGGCGTTAAGATCAAACCGGGTATGGTTATTGGCTATATCGTGCTGCGTGGTGACGGCCCGATTAGCAACCGTGCGATCCTGGCGGAGGAATACGACCCGAAAAAGCACAAATATGATGCGGAGTACTATATTGAAAACCAAGTTCTGCCGGCGGTGCTGCGTATCCTGGAGGGTTTTGGCTACCGTAAGGAAGATCTGCGTTATCAAAAGACCCGTCAAGTTGGCCTGACCAGCTGGCTGAACATCAAGAAAAGCTAA

## Sequence S3 The codon-optimized DNA sequence of CueO (amino acid residues 29–516)

GCTGAAAGGCCTACACTACCCATACCAGATTTACTGACCACGGATGCACGCAACCGCATCCAATTGACCATTGGTGCGGGCCAAAGCACCTTTGGTGGTAAGACCGCAACCACCTGGGGCTACAACGGCAACCTGCTGGGTCCGGCGGTTAAGCTGCAACGTGGTAAGGCTGTGACGGTGGATATTTATAACCAGCTGACCGAGGAAACCACGCTGCATTGGCATGGCCTCGAGGTACCGGGTGAAGTGGACGGCGGTCCGCAAGGTATCATCCCGCCGGGTGGTAAGCGCAGCGTTACCCTTAACGTTGACCAACCGGCGGCTACCTGTTGGTTCCACCCGCATCAGCACGGCAAAACCGGCCGTCAGGTTGCAATGGGTCTGGCAGGTCTGGTCGTGATTGAAGACGATGAGATTTTGAAATTGATGTTGCCGAAACAGTGGGGTATCGACGACGTCCCGGTCATAGTGCAGGACAAAAAATTCAGCGCGGATGGCCAAATTGACTACCAATTGGATGTTATGACCGCAGCGGTGGGCTGGTTCGGTGACACATTGTTAACGAACGGCGCGATCTACCCGCAGCATGCTGCTCCGCGTGGCTGGCTGCGTCTGCGCCTGTTGAACGGGTGCAATGCAAGAAGCTTGAACTTCGCCACCAGCGACAACCGTCCGCTGTATGTTATCGCCTCCGATGGTGGCCTGTTGCCTGAACCGGTGAAGGTGAGCGAGCTGCCGGTGCTGATGGGTGAGCGCTTTGAAGTGCTGGTGGAAGTAAATGATAATAAGCCGTTCGACCTGGTTACCCTGCCAGTATCGCAAATGGGCATGGCGATCGCGCCATTTGACAAACCGCATCCGGTTATGCGTATCCAGCCGATCGCCATCTCGGCGTCAGGCGCGCTGCCGGATACCCTGAGCAGCCTGCCGGCGCTGCCCTCTCTTGAGGGTCTGACTGTTCGTAAACTTCAGCTGTCTATGGATCCGATGCTGGACATGATGGGTATGCAGATGCTGATGGAAAAATACGGCGACCAGGCAATGGCCGGTATGGACCACAGCCAGATGATGGGCCACATGGGTCATGGGAATATGAACCACATGAATCACGGCGGAAAGTTCGACTTCCACCACGCTAATAAGATCAATGGCCAAGCGTTTGACATGAACAAGCCGATGTTCGCTGCGGCGAAAGGTCAATATGAACGTTGGGTTATTTCCGGTGTTGGTGATATGATGCTGCACCCGTTTCATATTCATGGAACCCAGTTTCGTATTCTGTCCGAGAACGGTAAGCCGCCAGCCGCGCATCGTGCTGGCTGGAAAGATACTGTGAAGGTCGAGGGCAATGTTTCTGAGGTCTTGGTTAAGTTCAACCACGATGCCCCTAAAGAACACGCGTATATGGCGCACTGCCATCTCTTGGAGCACGAAGATACTGGTATGATGCTGGGCTTTACCGTGTGA
